# Manipulation of the degradation behavior of calcium sulfate by the addition of bioglass

**DOI:** 10.1007/s40204-019-0116-7

**Published:** 2019-05-24

**Authors:** Pei-Yi Hsu, Hsiao-Chun Kuo, Wei-Hsing Tuan, Shao-Ju Shih, Makio Naito, Po-Liang Lai

**Affiliations:** 1 Department of Materials Science and Engineering, National Taiwan University, Taipei, 107 Taiwan; 2 Department of Materials Science and Engineering, National Taiwan University of Science and Technology, Taipei, 107 Taiwan; 3 Joining and Welding Research Institute, Osaka University, 11-1, Mihogaoka, Ibaraki, Osaka 567-0047 Japan; 4 Department of Orthopedic Surgery, Bone and Joint Research Center, Chang Gung Memorial Hospital at Linkou, College of Medicine, Chang Gung University, Taoyuan, 333 Taiwan

**Keywords:** Composite, Bioceramic, Calcium sulfate, Bioglass, Degradation

## Abstract

A bioactive calcium sulfate/glass composite was prepared using a sintering technique, and Ca–P–Si glass particles were prepared by spray pyrolysis. The glass exhibited bioactivity in terms of its ability to form apatite in a simulated body fluid. The glass was transformed into two crystallized phases, i.e., calcium phosphate and calcium silicate, respectively, during the heating stage. The presence of the crystallized phases retarded the densification of calcium sulfate. A high sintering temperature of 1200 °C was needed to prepare the composite. The increased addition of glass enhanced the strength and decreases the degradation rate of calcium sulfate. The new composite is not only degradable but also bioactive.

## Introduction

Although autografts exhibit better biocompatibility compared to synthetic bioceramics, degradable bioceramics serve as alternatives for patients who want to avoid undergoing further donor site injury. Thus, several synthetic bioceramics are being developed, mainly including calcium salts, such as phosphate (Dorozhkin 2015), carbonate (Koroleva et al. 2012), sulfate (Nilsson et al. 2002), or silicate (Wu and Zreiqat 2007), or a mixtures of two salts (LeGeros et al. 1992). By releasing calcium ions after implantation, these degradable bioceramics aid in the recovery of bone defects (Rahaman et al. 2011).

Among these bioceramics, calcium sulfate and bioactive glass exhibit several desirable biological properties (Chen et al. 2005; Hench and Wilson 1984). Calcium sulfate exhibits biocompatibility and biodegradability, as well as an excellent capability for bone fusion (Chen et al. 2005). Considering bioactive glass, it could form a strong bond with osseous tissue and positively affect osteogenesis (Hench and Wilson 1984). Nevertheless, the degradation behavior of calcium sulfate and bioglass is significantly different. Calcium sulfate exhibits a rapid resorption rate, which is sometimes unpredictable (Thomas and Puleo 2009). On the other hand, bioactive glass typically exhibits a slow degradation rate (Islam et al. 2017). A mixture of calcium sulfate and bioactive glass may allow for the tailoring of their respective degradation behaviors.

The combination of these two bioceramics has attracted attention previously. Camargo et al. (2000) have demonstrated that bioactive glass and calcium sulfate are well tolerated within human oral tissue. As the barrier for a bioactive glass graft, calcium sulfate exhibits good performance for the preservation of the alveolar ridge after tooth extraction. Melo et al. (2005) have carried out tests on mixtures of calcium sulfate and bioactive glass using a rat tibial model. The rapid resorption rate of calcium sulfate induces the early ingress of osteoprogenitor cells. The slow degradation rate of the bioactive glass graft leads to a decreased amount of bone formation due to its continued presence. Silveira et al. (2008) have also applied calcium sulfate as a barrier for bioactive glass. This combination exhibits a better osteoconductivity: 24% bone formation in the central region is observed, which is greater than the value of 21% obtained using only calcium sulfate and that of 5% using only bioactive glass. All these studies have demonstrated considerable potential for the simultaneous use of calcium sulfate and bioactive glass.

In this study, a composite comprising calcium sulfate and bioactive glass is prepared by a sintering technique. Kuo et al. have investigated the sintering behavior of calcium sulfate (Kuo et al. 2012). The densification of calcium sulfate possibly occurs at 1100 °C. The sintering of bioactive glass can be carried out at a temperature lower than 1100 °C (Boccaccini et al. 2007). The presence of bioactive glass might provide bond at grain boundary, enhancing the strength of calcium sulfate (Shuai et al. 2015). However, the crystallization of glass possibly occurs at elevated temperatures. In this study, a dense calcium sulfate/glass composite is prepared through a sintering technique. In addition, the degradation behavior of the composite is examined.

## Materials and methods

### Preparation of bioactive glass particles

Spray pyrolysis was employed to prepare glass particles using tetraethyl orthosilicate [TEOS, Si(OC_2_H_5_)_4_, ACROS Organic Co., USA], calcium nitrate tetrahydrate [Ca(NO_3_)_2_·4H_2_O, Showa Co., Japan], and triethyl phosphate [(OC_2_H_5_)_3_PO_4_, Alfa Aesar, USA] as the precursors, which were adjusted, such that a SiO_2_:CaO:P_2_O_5_ ratio of 60 mol%:35 mol%:5 mol% was achieved. First, TEOS was dissolved in ethanol, while calcium nitrate tetrahydrate and triethyl phosphate were dissolved in water. These two solutions were mixed and stirred for 24 h. An ultrasonic nebulizer at 1.65 MHz was utilized to disperse the solution into small droplets, which were passed through a tube furnace at 700 °C. The resulting powders were negatively charged by using a tungsten filament at 16 kV and neutralized in an earthed stainless steel collector.

### Preparation of calcium sulfate/glass composites

Various amounts, i.e., 0 wt%, 1 wt%, 5 wt%, and 10 wt%, of the glass powder were mixed into a calcium sulfate hemi-hydrate powder (CaSO_4_·0.5 H_2_O, J.T. Baker, USA). The size of the calcium sulfate hemi-hydrate powder was characterized using a zeta-potential analyzer (Malvern, USA). Mixing was carried out in 99.5% ethyl alcohol for 4 h. Zirconia balls with a diameter of 10 mm were used as the milling media. After drying, the powder mixture was sieved through a #150 plastic mesh. The specimen pellets were prepared using a die-pressing technique. The applied uniaxial pressure was 30 MPa. The diameter and thickness of the green pellet were 10 mm and 3.8 mm, respectively. The pellets were sintered at temperatures ranging from 800 to 1200 °C. At the sintering temperature, the dwell time was 1 h.

### Characterization of the composite

Scanning electron microscopy (SEM, Jeol Co., Japan) and transmission electron microscopy (TEM, FEI Co., USA) were used for microstructure observation. A focused ion beam (FIB, FEI Co., USA) was utilized to reveal the cross section of the glass particles. The glass particle composition was analyzed by electron dispersive spectroscopy (EDS, Mahwah Co., USA). An X-ray diffractometer (XRD, Rigaku Co., Japan) was utilized to characterize the crystalline phase. To measure compressive strength, larger sized pellets were also prepared. The diameter and thickness of the pellet were 13 mm and 13 mm, respectively. A universal testing machine (MTS Co., USA) was used, and a displacement rate of 0.16 mm/s was applied.

### In vitro tests

The extract from the calcium sulfate/1% glass composites was used to evaluate the cell viability. The MC3T3-E1 cells were cultured in α-minimum essential medium (α-MEM, Invitrogen Corp.), comprised of 10% fetal bovine serum (FBS; Gibco-BRL), 100 μg/mL penicillin, 100 U/mL streptomycin, 250 ng/mL fungizone, and 50 μg/mL gentamycin (Gibco-BRL), for 3 days, followed by extract collection. The cells were loaded into the extract at a density of 2 × 10^4^ cells/well. Cell viability was estimated after 1 day using a microplate reader (Infinite 200 PRO, Tecan Co., Switzerland) at a wavelength of 450 nm. The test was conducted in triplicate.

The degradation test of the calcium sulfate/glass composites was carried out in a simulated body fluid and a phosphate buffered saline (PBS, Gibco, USA) solution. The ISO-10993-14 protocol was applied. First, the pellets were dried and weighed, followed by soaking in the PBS solution at a ratio of 1 (g) to 10 (mL). After 24 h, the pellets were weighed again. After soaking the pellets in the PBS solution, the solution was collected. The amount of calcium, phosphate, and silicon ions in the PBS solution was determined by inductively coupled plasma-mass spectroscopy (ICP-MS, Perkin Elmer Co., USA). The PBS solution was then refreshed on a daily basis, and this process was repeated 28 times.

## Results

### Bioactive glass particles

**Fig. 1:**
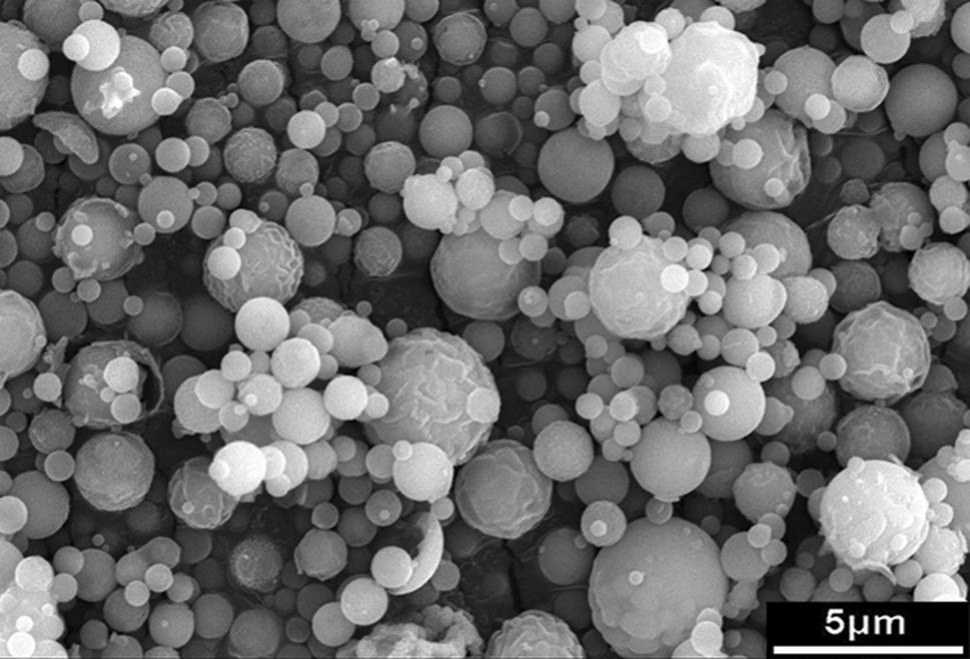
Morphology of the glass particles prepared by spray pyrolysis

**Fig. 2:**
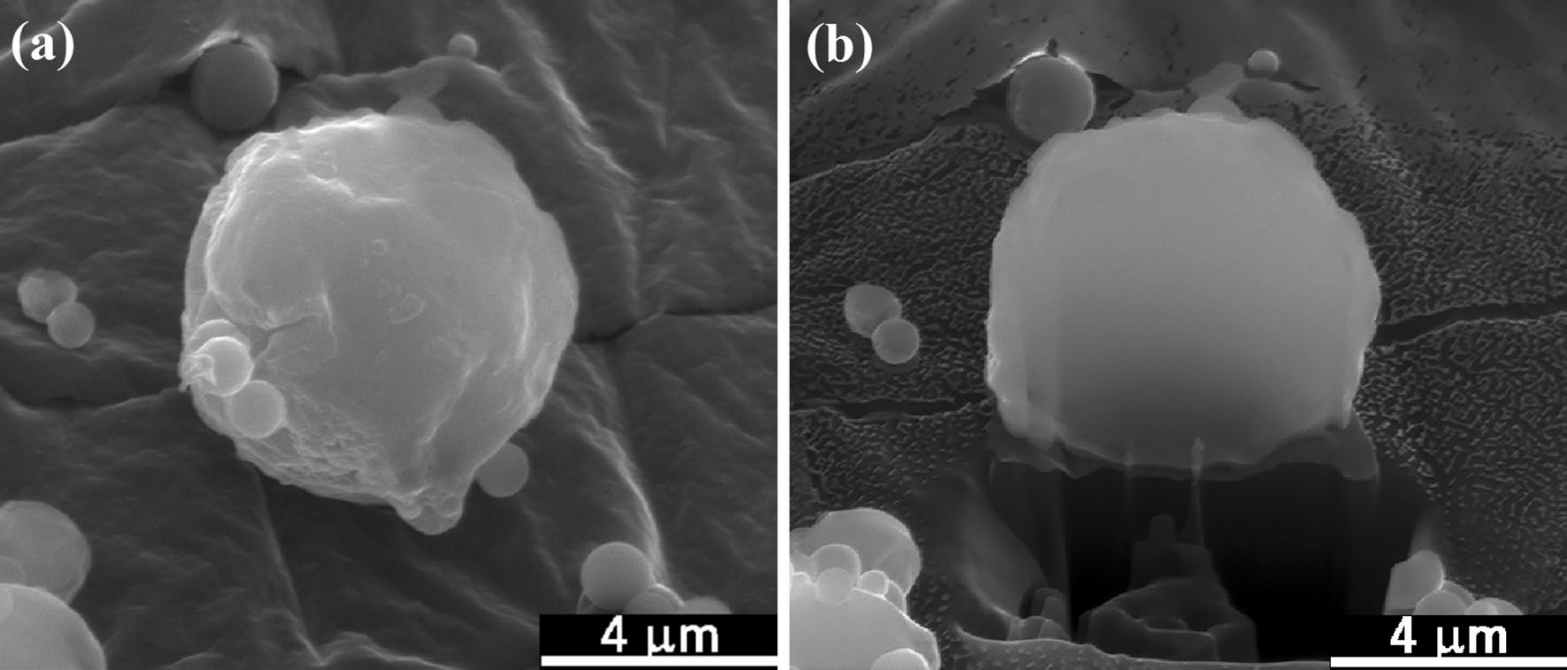
**a** Morphology of one glass particle and **b** its cross section exposed by the FIB technique

Figure 1 shows the morphology of the glass particles prepared by spray pyrolysis: Spherical particles with sizes varying from 0.3 to 2.6 μm, with a mean particle size of 1 μm, are observed. Figure 2a shows the morphology of a typical glass particle and its cross section (Fig. 2b). Pores are not observed within the particle, indicative of a solid particle in the core. The EDS analysis of the cross section revealed Si, Ca, and P contents of 57.1 mol%, 32.7 mol%, and 10.2 mol%, respectively. This composition corresponds to a ratio of SiO_2_/CaO/P_2_O_5_ as 57/31/12 wt%. These values are similar to the composition of the starting solution.

**Fig. 3:**
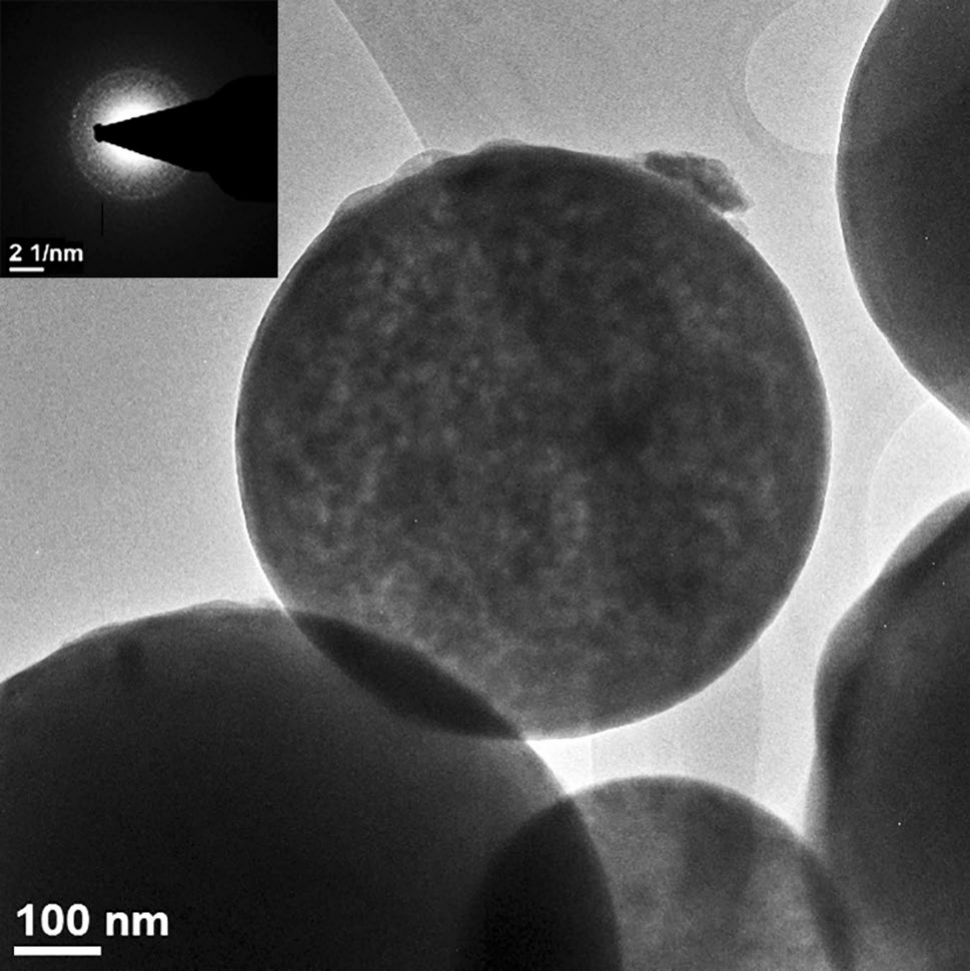
TEM image and the corresponding SAED pattern (inset) of the glass particle

**Fig. 4:**
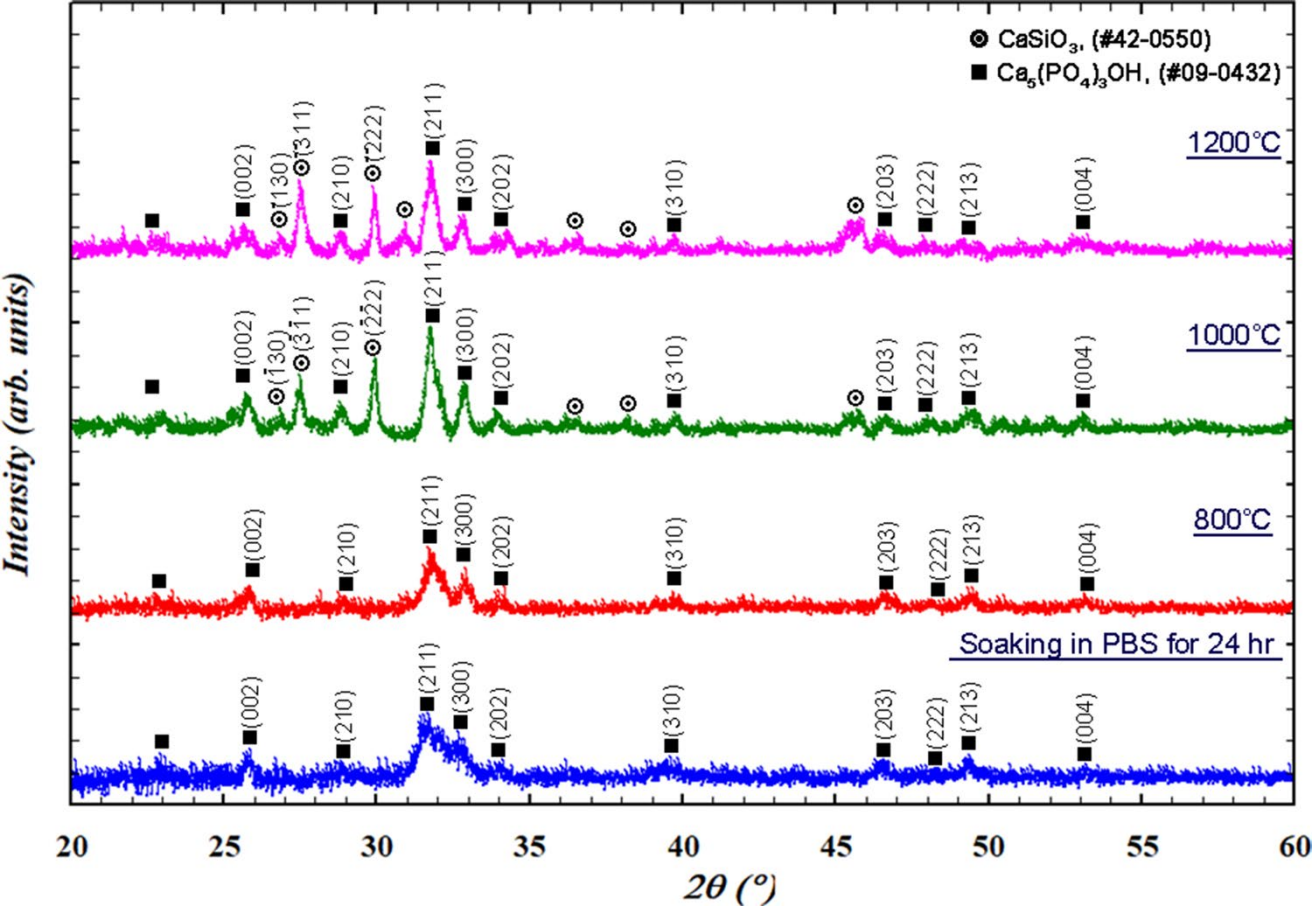
XRD patterns of the glass after soaking in PBS for 24 h and after firing at 800 °C, 1000 °C, and 1200 °C

Figure 3 shows the TEM micrograph of the glass particles, which also confirmed the presence of solid glass particles inside the core. The selected-area electron diffraction (SAED) pattern (inset in Fig. 3) revealed a predominantly amorphous particle, albeit with minor nanocrystals. To evaluate the bioactivity of the glass particles, the glass powder was soaked in a PBS solution for 24 h. Figure 4 shows the XRD pattern of the glass powder after soaking. A small amount of apatite (calcium phosphate) was detected.

### Calcium sulfate/glass composites

**Fig. 5:**
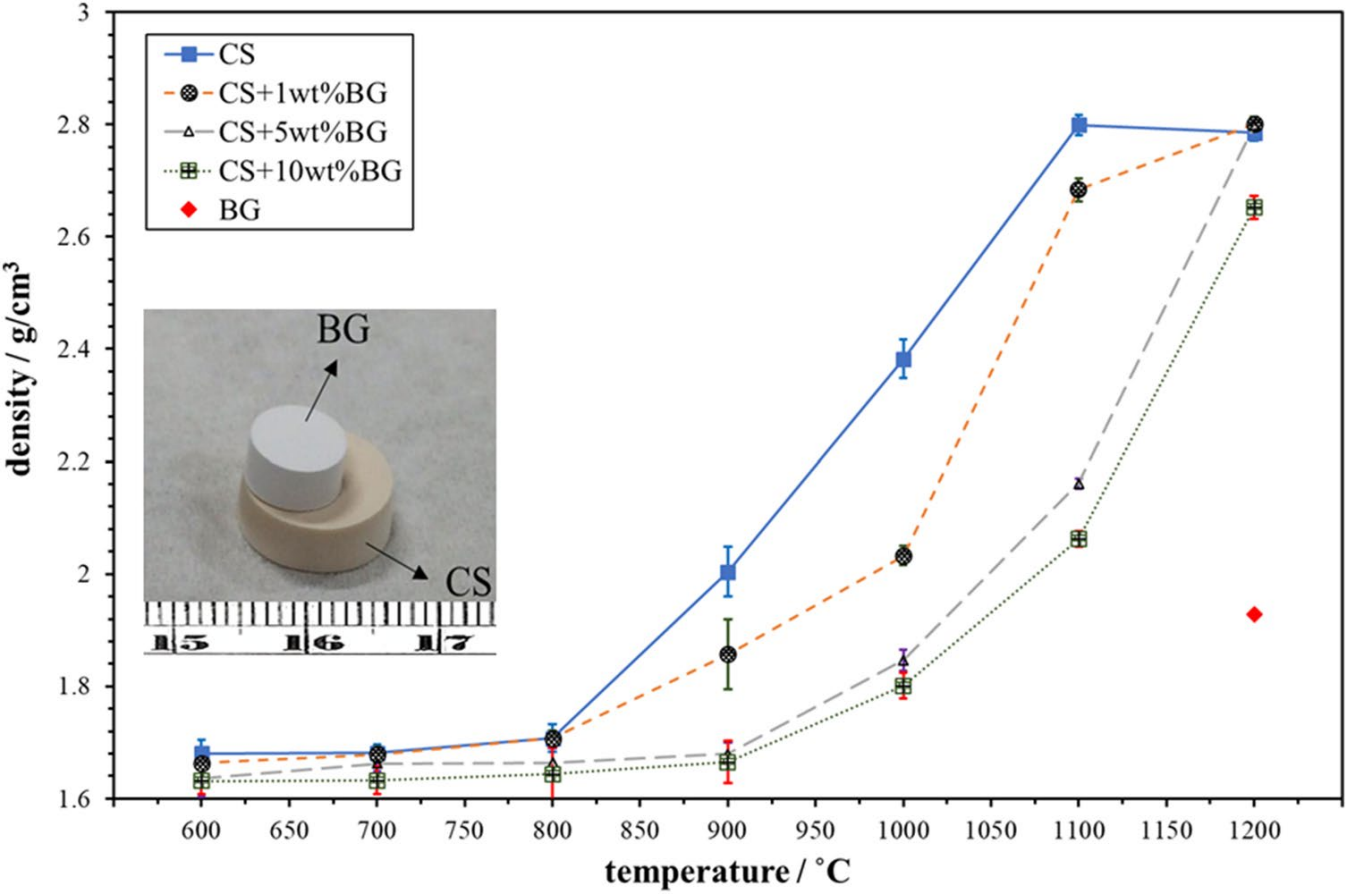
Density of the calcium sulfate (CS)/glass (BG) composites as a function of sintering temperature. Also shown is the density of the glass pellet after sintering at 1200 °C (red diamond). Inset shows the morphology of a glass pellet on the top of a calcium sulfate specimen after sintering to 1200 °C

In this study, calcium sulfate matrix composites are prepared by a sintering technique. Before sintering, the phase stability of the glass at an elevated temperature is first investigated. Figure 4 also shows the XRD patterns of the glass after heat treatment at 800 °C, 1000 °C, and 1200 °C for 1 h. The calcium phosphate phase is observed after heating at 800 °C. As the firing temperature was increased, the XRD peaks become sharper. The formation of a reaction phase, i.e., calcium silicate (CaSiO_3_), starts at a temperature greater than 1000 °C. After heating to 1200 °C, both calcium phosphate and calcium silicate are observed. The density of the glass pellet after sintering at 1200 °C is 1.9 g/cm^3^ (Fig. 5); this value is less than the theoretical densities of hydroxyapatite (3.63 g/cm^3^) and calcium silicate (2.9 g/cm^3^).

The mean particle size of the calcium sulfate powder is 0.57 μm. Calcium sulfate particles are slightly smaller than the glass particles. Figure 5 shows the density of the calcium sulfate/glass composite as a function of the sintering temperature. Pure calcium sulfate attains its highest density after heating to 1100 °C. The addition of glass particles decreases the density of calcium sulfate. The densities of composites with a glass content from 1 to 10 wt% glass are similar after heating at 1200 °C. Hence, this temperature is utilized to prepare the calcium sulfate/glass composites for further characterization.

### Characterization of composites

**Fig. 6:**
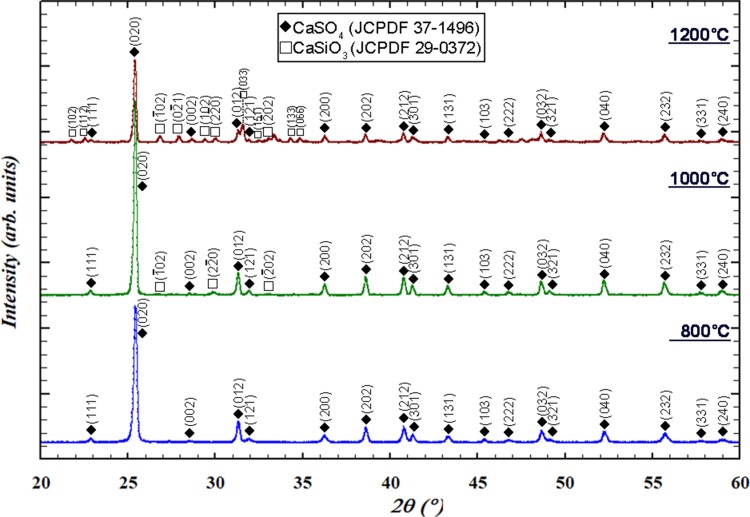
XRD patterns of the calcium sulfate/10% glass composite after sintering at various temperatures

**Fig. 7:**
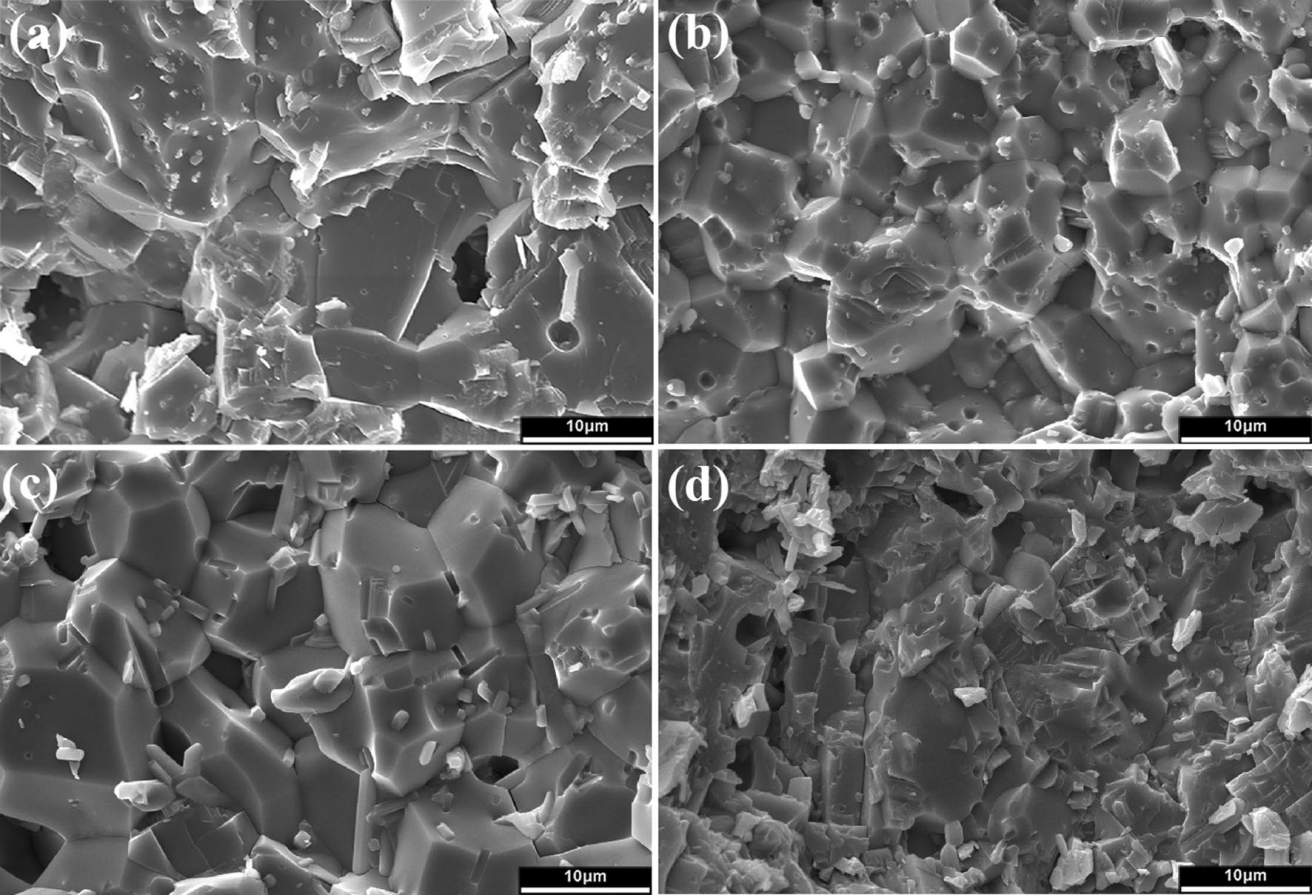
Fracture surfaces of the calcium sulfate/glass composites. The content of glass in the composites is **a** 0%, **b** 1%, **c** 5%, and **d** 10%

Figure 6 shows the XRD patterns of the calcium sulfate/10% glass composite after heat treatment. After heating to 800 °C, the calcium sulfate hemi-hydrate is transformed to calcium sulfate anhydrite. The reaction phase, calcium silicate (CaSiO_3_), is detected starting at 1000 °C and the amount slightly increases with temperature. Calcium phosphate is not detected possibly due to its low content. Figure 7 shows the fracture surfaces of the composites. The composites were prepared by sintering at 1200 °C. The grain size of pure calcium sulfate is greater than 10 μm. In the composites, fine particles are observed at the boundaries between the calcium sulfate grains. The size of calcium sulfate grains is smaller because of the presence of these fine particles.

The compressive strength of the pure calcium sulfate is ~ 130 MPa (Table 1).

**Table 1:** Compressive strength for the calcium sulfate/glass composites

Composition (wt%)	Compressive strength (MPa)
Calcium sulfate	130 ± 20
Calcium sulfate (1% glass)	70 ± 18
Calcium sulfate (5% glass)	167 ± 5
Calcium sulfate (10% glass)	146 ± 6

The addition of 1 wt% glass leads to a decrease in the compressive strength of calcium sulfate by nearly half of its original value. However, with an increase in the glass content to 5 and 10 wt%, the compressive strength is enhanced. In the case of the composite containing 5 wt% glass, the compressive strength is 167 MPa; this value is ~ 30% greater than that of the calcium sulfate pellet.

### In vitro tests

**Fig. 8:**
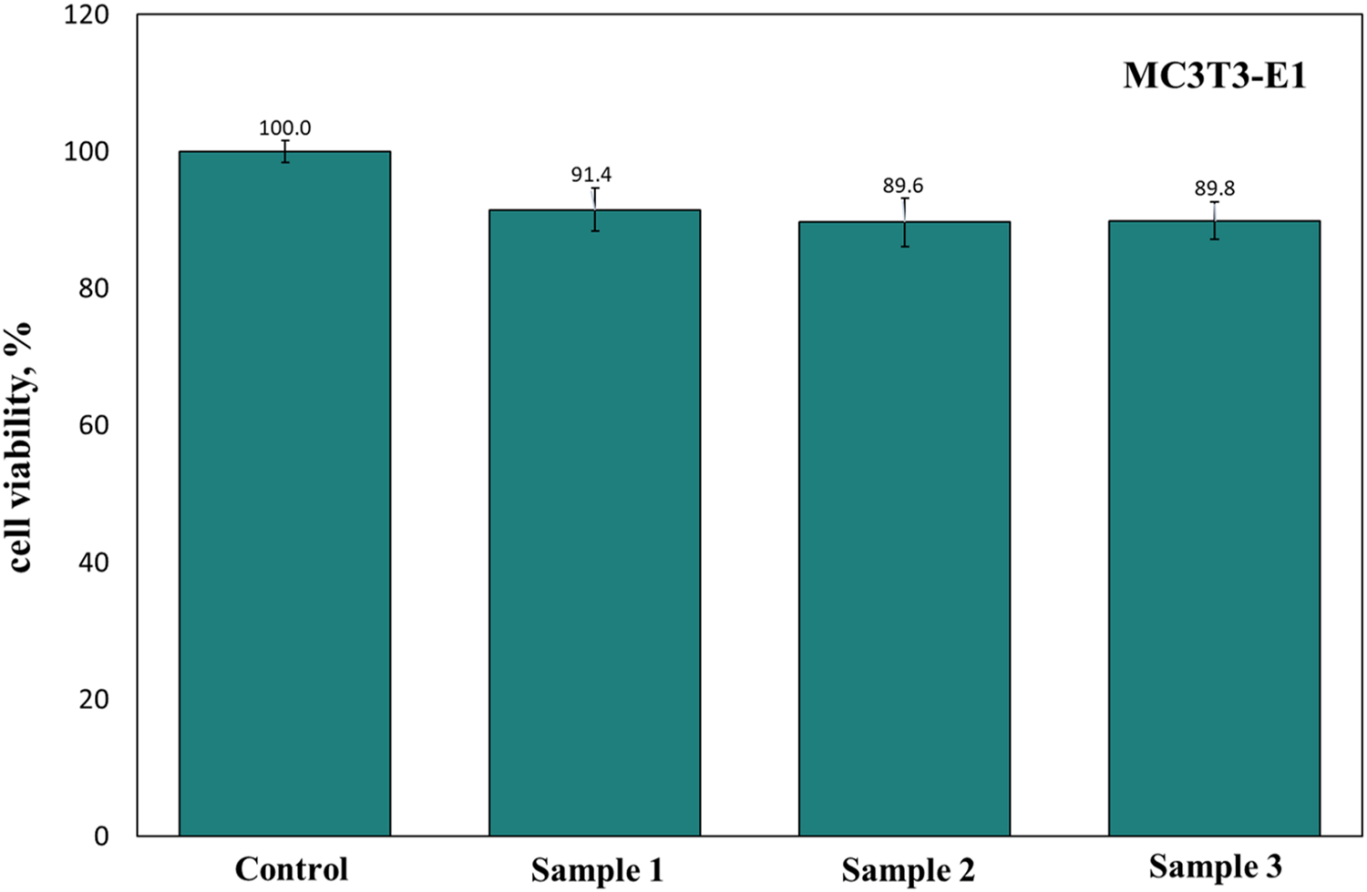
Viability of MC3T3-E1 cells in the extract from CSA/1% glass pellets

**Fig. 9:**
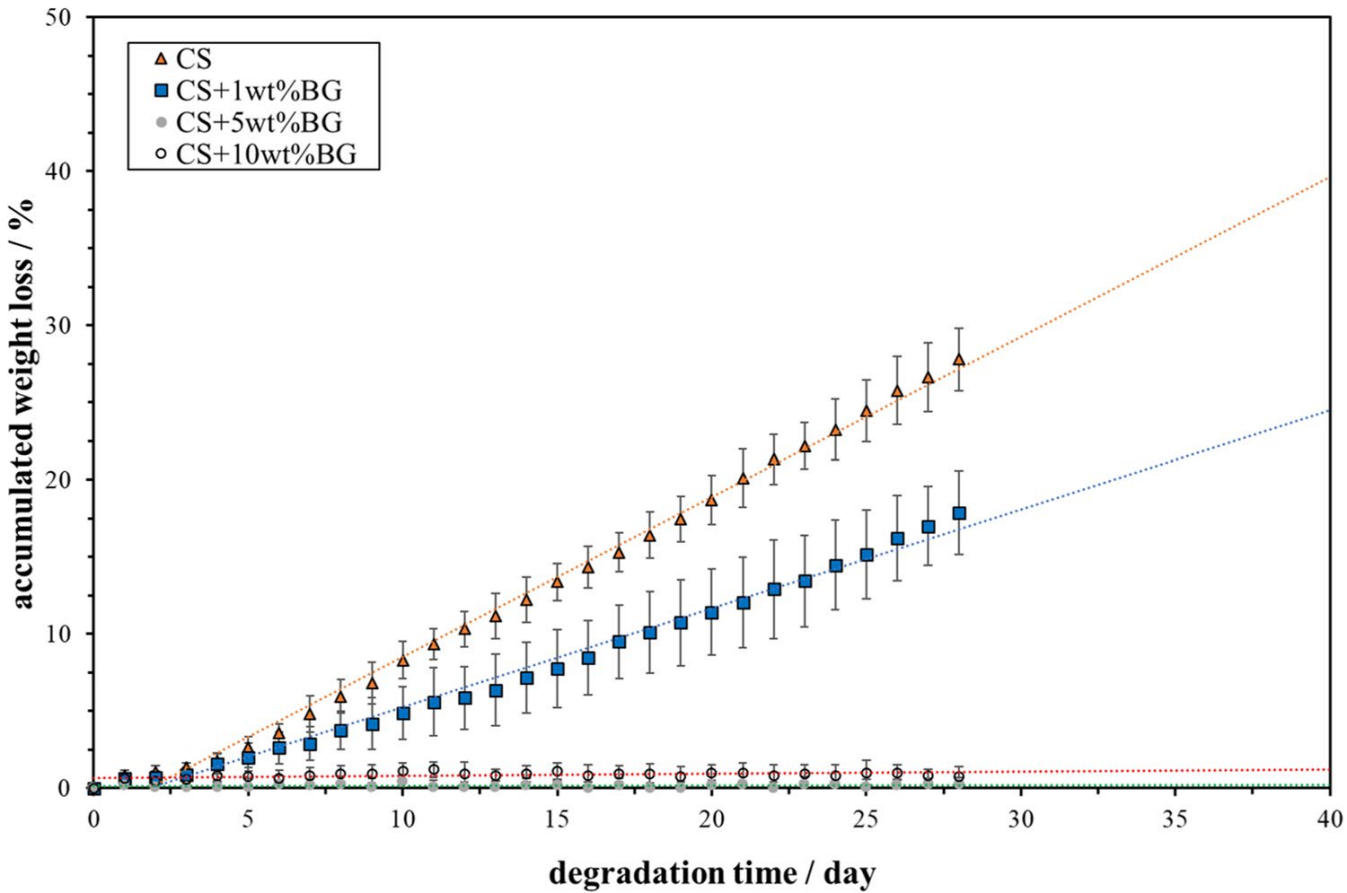
Accumulated weight loss for the calcium sulfate (CS)/glass (BG) composite in the PBS solution as a function of time

**Fig. 10:**
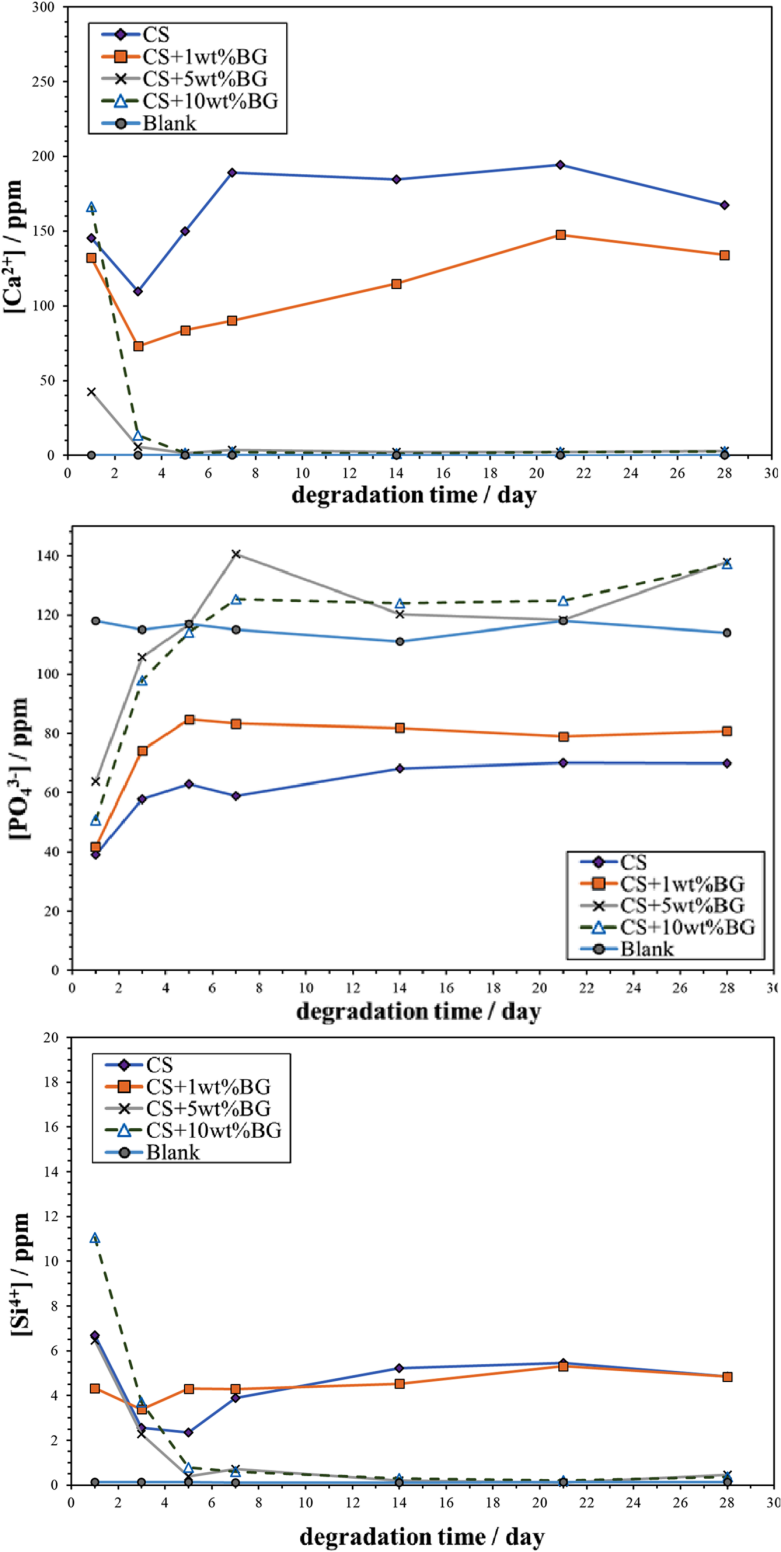
Concentrations of **a** Ca^2+^, **b** PO_4_^3−^, and **c** Si^4+^ in the PBS solution after soaking the calcium sulfate/glass composites. Also shown is the concentration of Ca^2+^, PO_4_^3−^, and Si^4+^ in the blank PBS solution

After incubating the cells in the extract from the composite for 1 day, the cell viability is greater than 90% (Fig. 8). An about 1% daily weight loss is observed for the calcium sulfate pellet in the PBS solution (Fig. 9). Furthermore, after soaking the pellet in the PBS solution for 28 days, its accumulated weight loss reaches 28%. With the addition of 1 wt% glass, the degradation rate decreases by ~ 40%. For the composites containing 5 wt% or 10 wt% glass, the accumulated weight loss of the composite is extremely low (~ 1%). Figure 10 shows the concentrations of Ca^2+^, PO_4_^3−^, and Si^4+^ in the PBS solution, as detected by ICP. In addition, the concentration of these ions in the blank PBS solution is determined by the same technique. In the blank PBS solution, the Ca^2+^ concentration is zero. Hence, Ca^2+^ is released from the pellets (Fig. 10a). The calcium sulfate pellet releases ~ 180 ppm Ca^2+^ into the PBS solution daily. The addition of 1% glass leads to decrease of this amount to ~ 100 ppm.

The concentration of PO_4_^3−^ in the blank PBS solution is ~ 120 ppm (Fig. 10b). The concentration of PO_4_^3−^ in the PBS solution after soaking pure calcium sulfate is ~ 60 ppm. For the composite containing 1 wt% glass, the concentration of PO_4_^3−^ in the PBS solution is ~ 80 ppm. In terms of the composites containing 5% and 10% glass soaked in the PBS solution, the amount of PO_4_^3−^ is similar to that in the blank PBS solution, ~ 120 ppm.

The calcium sulfate pellet releases ~ 5 ppm Si^4+^ into the PBS solution (Fig. 10c). As there is no silicon in the calcium sulfate specimen, the presence of Si^4+^ can be considered to be the background. The concentration of Si^4+^ in the PBS solution after soaking the composites containing 1–10 wt% glass is also similar to 5 ppm, indicative of the extremely slow release of Si^4+^ from calcium silicate.

**Fig. 11:**
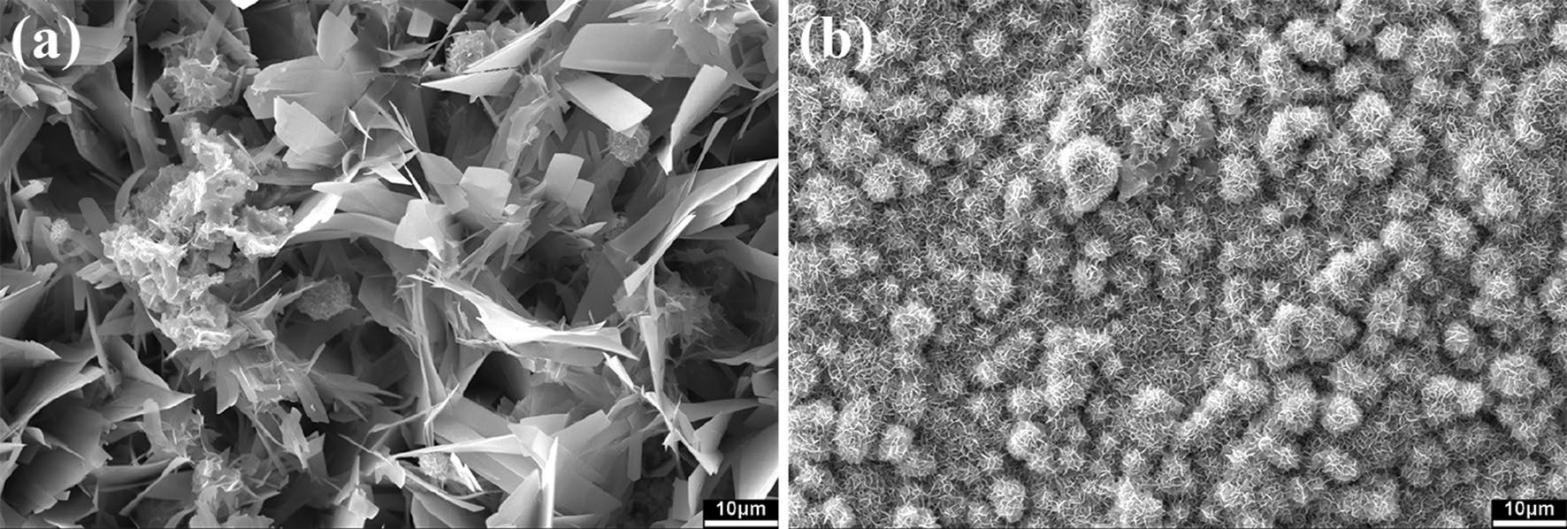
Surface morphology of the **a** calcium sulfate/1% glass and **b** calcium sulfate/10% glass composites after soaking in PBS for 28 days

**Fig. 12:**
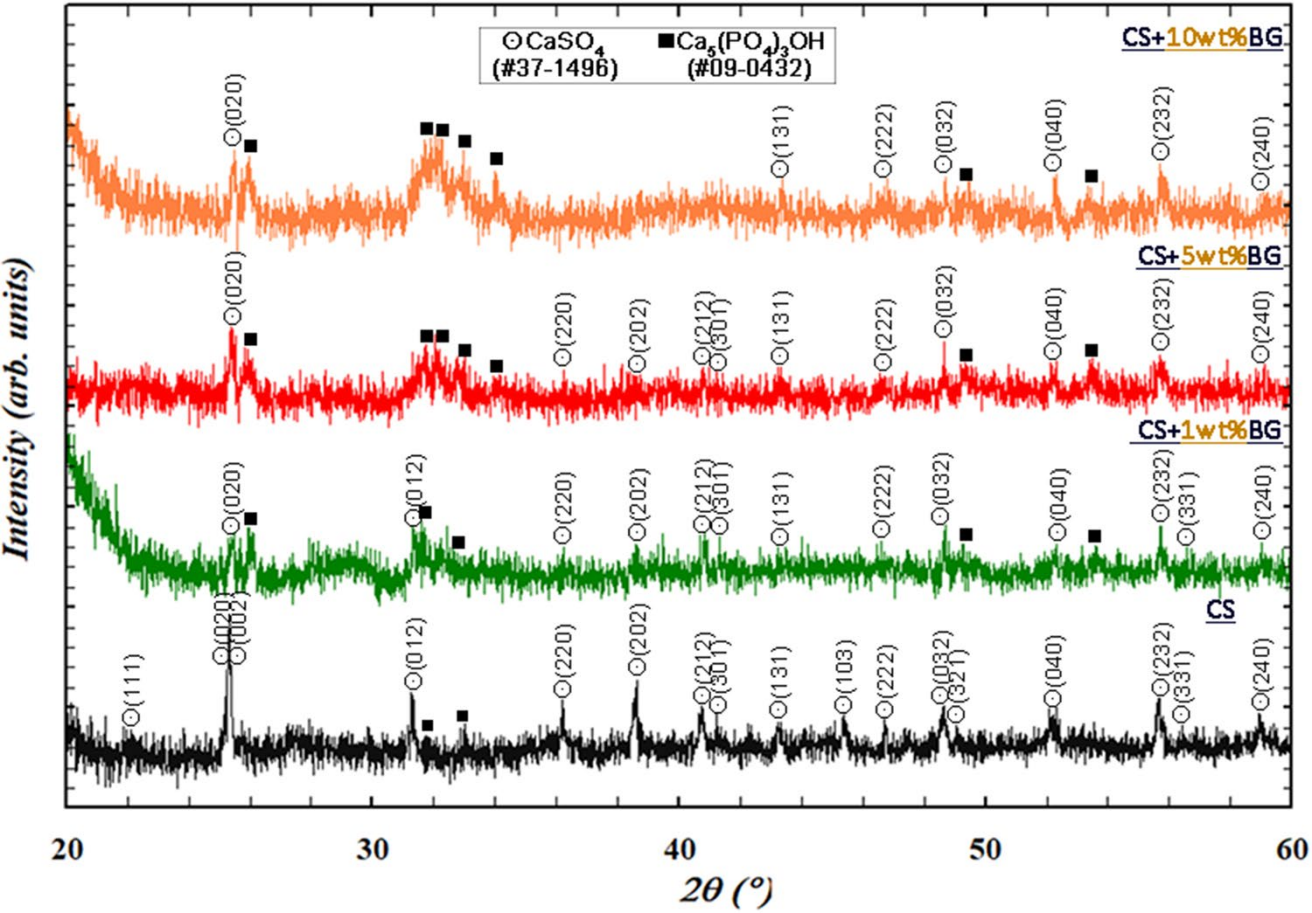
XRD patterns of the calcium sulfate (CS)/glass (BG) composites after soaking in PBS for 28 days

Figure 11 shows the surface morphology of the composites after soaking in the PBS solution for 28 days. Several flakes are observed on the surface of the composite containing 1 wt% glass (Fig. 11a). For the composite containing 10% glass, the size of flakes considerably decreases. Several clusters comprising small flakes are formed instead (Fig. 11b). As identified by XRD, the precipitate phase is calcium phosphate (Fig. 12). The amount of calcium phosphate increases with the increase of glass content.

## Discussion

In this study, spherical glass particles are prepared by spray pyrolysis (Fig. 1), which first generates liquid drops, leading to the formation of spherical particles after pyrolysis at 700 °C. The residence time of the drops in the pyrolysis furnace is extremely short; thus, the phase of the resulting particles is mainly amorphous. The calcium phosphate phase is observed after soaking the glass powder in a simulated body fluid (Fig. 4). The formation of the calcium phosphate phase is crucial for bonding to the bone tissue; its formation is considered to be an indication of bioactivity (Hench and Wilson 1984). Thus, the glass prepared by the sintering technique is bioactive.

The sintering of the glass pellet can afford a density of only 1.9 g/cm^3^ (Fig. 5). The glass is no longer viscous after crystallization at temperatures of greater than 800 °C (Fig. 4). The wetting of the glass pellet on the calcium sulfate pellet is no longer possible (inset of Fig. 5). The nucleation and growth of the crystalline phases of glass at elevated temperatures cannot be prevented. The formation of calcium phosphate begins at 800 °C, while that of calcium silicate begins at 1000 °C (Fig. 4). The transformation from glass to the crystallized phases involves a change in density. The density of completely dense silicate glass is ~ 2.5 g/cm^3^ (Roger et al. 2012). The theoretical densities of calcium phosphate, such as hydroxyapatite, and calcium silicate are greater than this value. Hence, the formation of a crystalline phase is detrimental to the densification of glass. However, the densification of the glass pellet is no longer a problem as the glass is used as the second phase of the composite.

The heat treatment of bioactive glass can lead to other possibilities. For example, sintering has been employed for preparing porous glass–ceramic scaffolds for tissue engineering applications (Boccaccini et al. 2007). For the glass prepared herein, the resulting crystalline phase after sintering includes calcium phosphate and calcium silicate; these two ceramics have been applied as biomaterials (Dorozhkin 2015; Wu and Zreiqat 2007). Nevertheless, some calcium phosphates, such as hydroxyapatite, are non-degradable; the degradation rate of calcium silicate is also slow (Chang et al. 2017).

The calcium sulfate/glass composites with a density greater than 2.7 g/cm^3^ are prepared by sintering at 1200 °C. The estimated relative density of the composites is ~ 93%. With such a high density, the pores are no longer interconnected within the composites. Hence, the degradation rate of the composites is slow. Furthermore, as the pores serve as the concentration sites for stresses (Rice 1977), a low porosity ensures high compressive strength. The compressive strength of sintered calcium sulfate can reach 130 MPa (Table 1). The compressive strength of the composites can be greater than 165 MPa as long as the glass content is greater than 5 wt%. Although the size of the glass particles (~ 1 μm) is similar to that of calcium sulfate particles (~ 0.57 μm), the mixing of calcium sulfate and an extremely small amount of the second phase is challenging. A minimum amount of 5 wt% is required to ensure a uniform microstructure. Calcium silicate particles are mainly located at the grain boundaries of calcium sulfate (Fig. 7). The microstructure of calcium sulfate is refined because of the presence of these particles. Hence, the compressive strength is enhanced.

**Fig. 13:**
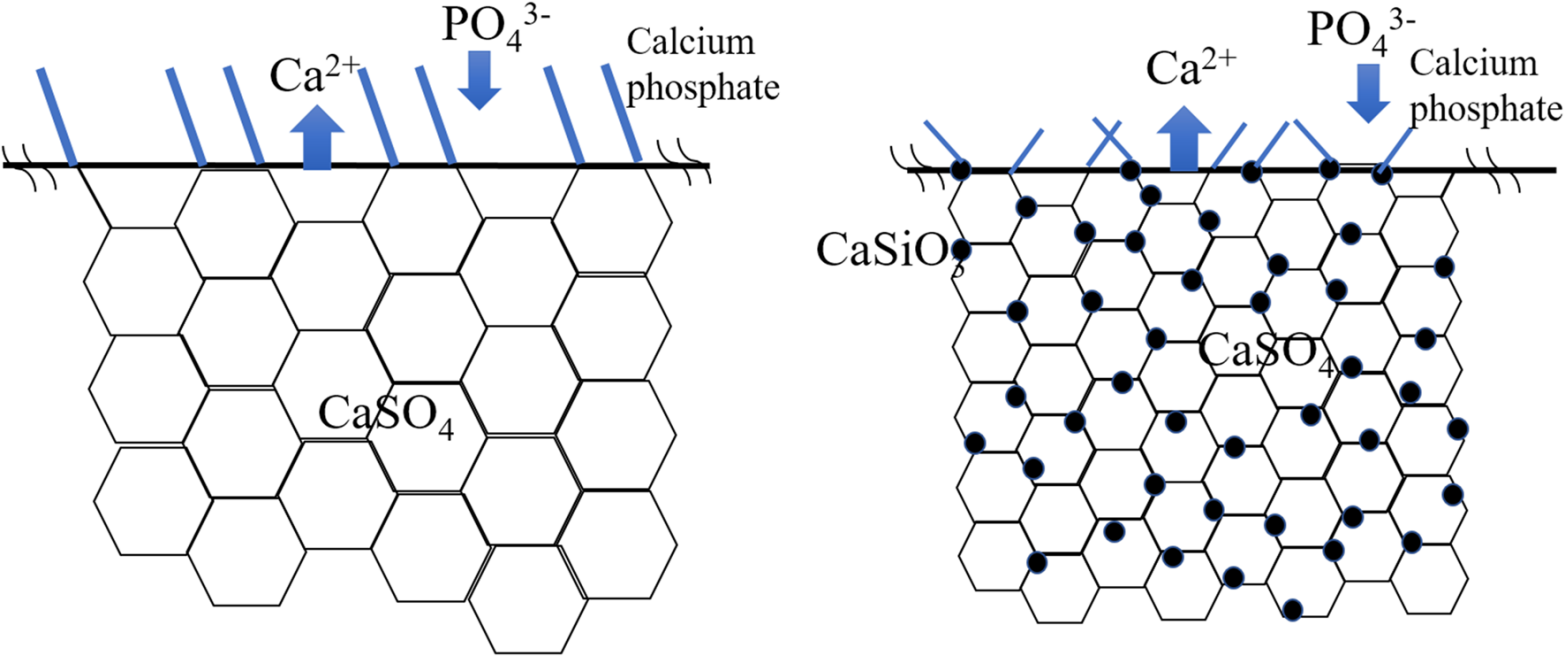
Schematic of surface ion-exchange process for **a** calcium sulfate and the **b** calcium sulfate composite. Calcium sulfate and its composite can release calcium ions and exchange with the phosphate ions in the solution, affording calcium phosphate flakes

With the increased addition of glass, the degradation rate significantly decreases (Fig. 9). The degradation is strongly related to the release of Ca^2+^ (Fig. 10a). The release of Ca^2+^ from the calcium sulfate pellet promotes the exchange of ions on its surface. The exchange between Ca^2+^ (from the composite) and PO_4_^3−^ (from the PBS solution) induces the surface formation of calcium phosphate. Figure 13 shows the schematic of the ion-exchange process, highlighting the mechanism for the surface formation of calcium phosphate. The size of the calcium phosphate crystals on the surface decreases with the increased addition of glass (Fig. 11). The calcium silicate particles within the composite may serve as nucleation sites for the formation of calcium phosphate on the surface (Fig. 13b). Thus, the increase in the amount of second-phase particles encourages the increase in the amount of calcium phosphate on the surface.

An extremely small amount of silicon ions may dissolve into calcium sulfate after sintering at elevated temperatures. Although the solubility of silicon in calcium sulfate is less than 0.1% (Chang et al. 2017), the addition of silicon ions leads to a significant decrease in the degradation rate of calcium sulfate. Hence, the release of Ca^2+^ is decreased after the addition of silicon-containing glass. The degradation of calcium silicate is slow (Chang et al. 2017), and the release of Si^4+^ is extremely low (Fig. 10c).

The bioactivities of bioceramics are key to their use in tissue engineering (Hench and Wilson 1984). The addition of the glass particles leads to an increase in not only the amount of the calcium phosphate phase on the composite surface but also the compressive strength (Fig. 12). Calcium phosphates constitute a large family comprising hydroxyapatite, tricalcium phosphate, dicalcium phosphate anhydrate, and octacalcium phosphate (Pietrzak 2008). The identification of each phase is challenging; nevertheless, most of these compounds are biocompatible. Their formation is beneficial for the use of these composites for future bone tissue engineering applications.

## Conclusions

In this study, a glass powder is prepared by spray pyrolysis. Apatite is formed after soaking the glass powder in a simulated body fluid, indicative of bioactive glass. Subsequently, the glass powder is mixed with calcium sulfate, affording a powder mixture. Then, a sintering technique is employed to prepare a dense calcium sulfate/glass composite. To prepare a composite with high compressive strength, a minimum amount, i.e., 5 wt%, is required. Nevertheless, a low amount, i.e., 1 wt%, of glass can decrease the degradation rate of calcium sulfate by 40%. The composite releases calcium ions into the simulated body fluid to exchange with the phosphate ions present in the fluid, affording a calcium phosphate surface layer after soaking the composite in the simulated body fluid.
